# (*R*_P_)-Menthyl (1-hydroxy­cyclo­hexyl)phenyl­phosphinate

**DOI:** 10.1107/S1600536810009219

**Published:** 2010-03-17

**Authors:** Baoci Fu, Chang-Qiu Zhao

**Affiliations:** a College of Chemistry and Chemical Engineering, Liaocheng University, Shandong 252059, People’s Republic of China

## Abstract

The title compound, C_22_H_35_O_3_P, features a tetra­hedral P atom bonded to a phenyl ring, a hydroxy­cyclo­hexyl unit and the O atom of a menthyl group. The axial chirality at phospho­rus is *R*_P_. In the crystal, mol­ecules are connected through O—H⋯O hydrogen bonds involving the hydr­oxy and P=O groups, forming chains along the 2_1_ screw axis. The methyl groups of the isopropyl fragment in the menthyl unit are disordered over two sites of equal occupancy.

## Related literature

For general background to α-hydr­oxy alkyl­phospho­nates, see: Kim & Wiemer (2003 ▶). For the structures of related phenyl­phosphinates, see: Sheldrick *et al.* (1981 ▶); Chaloner *et al.* (1991 ▶); Grice *et al.* (2004 ▶).
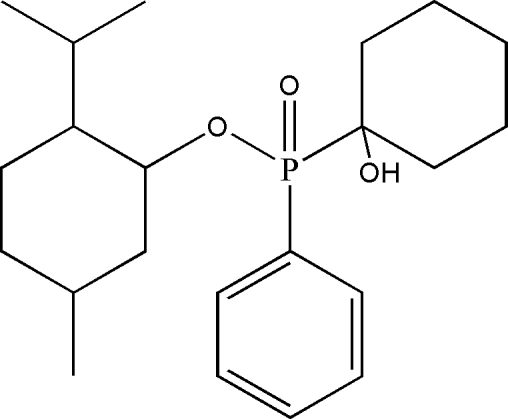


## Experimental

### 

#### Crystal data

C_22_H_35_O_3_P
*M* *_r_* = 378.47
Monoclinic, 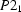

*a* = 10.1808 (11) Å
*b* = 11.0611 (13) Å
*c* = 10.4207 (12) Å
β = 106.201 (1)°
*V* = 1126.9 (2) Å^3^
*Z* = 2
Mo *K*α radiation
μ = 0.14 mm^−1^
*T* = 298 K
0.42 × 0.32 × 0.26 mm

#### Data collection

Siemens SMART CCD area-detector diffractometer
Absorption correction: multi-scan (*SADABS*; Sheldrick, 1996 ▶) *T*_min_ = 0.944, *T*_max_ = 0.965
5667 measured reflections
3787 independent reflections
3248 reflections with *I* > 2σ(*I*)
*R*_int_ = 0.019

#### Refinement

*R*[*F* ^2^ > 2σ(*F* ^2^)] = 0.039
*wR*(*F* ^2^) = 0.096
*S* = 1.06
3787 reflections
263 parameters
1 restraint
H atoms treated by a mixture of independent and constrained refinement
Δρ_max_ = 0.16 e Å^−3^
Δρ_min_ = −0.24 e Å^−3^
Absolute structure: Flack (1983 ▶), 1685 Friedel pairs
Flack parameter: 0.14 (10)

### 

Data collection: *SMART* (Siemens, 1996 ▶); cell refinement: *SAINT* (Siemens, 1996 ▶); data reduction: *SAINT*; program(s) used to solve structure: *SHELXS97* (Sheldrick, 2008 ▶); program(s) used to refine structure: *SHELXL97* (Sheldrick, 2008 ▶); molecular graphics: *SHELXTL* (Sheldrick, 2008 ▶); software used to prepare material for publication: *SHELXTL*.

## Supplementary Material

Crystal structure: contains datablocks I, global. DOI: 10.1107/S1600536810009219/bh2267sup1.cif

Structure factors: contains datablocks I. DOI: 10.1107/S1600536810009219/bh2267Isup2.hkl

Additional supplementary materials:  crystallographic information; 3D view; checkCIF report

## Figures and Tables

**Table 1:** Hydrogen-bond geometry (Å, °)

*D*—H⋯*A*	*D*—H	H⋯*A*	*D*⋯*A*	*D*—H⋯*A*
O3—H3⋯O2^i^	0.76 (3)	1.94 (3)	2.695 (3)	170 (3)

Symmetry code: (i) 
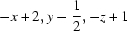
.

**Fig. 1.**
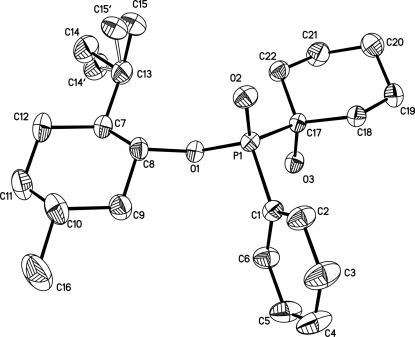
The molecular structure of the title compound. H atoms have been omitted for clarity. Primed atoms C14' and C15' are disordered with C14 and C15, respectively.

## supplementary crystallographic information

### Comment

α-Hydroxy alkylphosphonates have received attention both as substrates for the preparation of other α-substituted phosphonates, and because of their potential biological activity. For example, representatives of this class act as inhibitors of farnesyl protein transferase (FPTase), renin, and HIV protease (Kim & Wiemer, 2003).

The P-chiral title compound, which can be synthesized by addition of (*R*_P_)-menthyl-phenylphosphinate to cyclohexanone (see *Experimental*), is comprised of fully extended substituents: phenyl, menthyl and α-hydroxycyclohexyl. The configuration of the central P atom is *R* and the four groups around the P atom form an irregular tetrahedron as found in *tert*-butyl diphenylphosphinate (Grice *et al.*, 2004). The bond angles are C1—P—C17 = 107.13 (11)°, O1—P—C1 = 107.06 (10)°, O1—P—C17 = 102.87 (9)°, O2—P—O1 = 113.87 (9)°, O2—P—C17 = 113.18 (11)° and O2—P—C1 = 112.05 (11)°, which compare with angles observed in related phenylphosphinate derivatives bearing a menthyl group (Chaloner *et al.*, 1991; Sheldrick *et al.*, 1981). Part of methyl groups (C14 and C15) were found to be disordered over two sites with equal occupancies.

Intramolecular O3—H3···O2 hydrogen bonds are found in the crystal structure. The crystal packing is further stabilized by van der Waals interactions.

### Experimental

Cyclohexanone was added to a stirred DMF solution of (*R*_P_)-menthyl-phenylphosphinate in a flask and the mixture was stirred for 48 h at room temperature. After washing with water, the resulting solid was dried, and recrystallized from diethyl ether, to afford the pure title product.

### Refinement

Atoms C14 and C15 were found to be disordered over two sites, and the ratio of the occupancy factors was fixed to 0.50:0.50 and 0.50:0.50 for atoms C14:C14'and C15:C15', respectively. All H atoms attached to C atoms were fixed geometrically and treated as riding with C—H = 0.93–0.98 Å, and with *U*_iso_(H) = 1.2 *U*_eq_(C) or 1.5 *U*_eq_(C) for the methyl groups. Atom H3 was found in a difference map and refined with free coordinates, converging to O—H = 0.76 (3) Å. Assignment of absolute configuration is based on measurement of 1685 Friedel pairs.

### Crystal data

**Table:**

C_22_H_35_O_3_P	*F*(000) = 412
*M**_r_* = 378.47	*D*_x_ = 1.115 Mg m^−^^3^
Monoclinic, *P*2_1_	Mo *K*α radiation, λ = 0.71073 Å
Hall symbol: P 2yb	Cell parameters from 2598 reflections
*a* = 10.1808 (11) Å	θ = 2.5–24.8°
*b* = 11.0611 (13) Å	µ = 0.14 mm^−^^1^
*c* = 10.4207 (12) Å	*T* = 298 K
β = 106.201 (1)°	Block, colorless
*V* = 1126.9 (2) Å^3^	0.42 × 0.32 × 0.26 mm
*Z* = 2	

### Data collection

**Table:**

Siemens SMART CCD area-detector diffractometer	3787 independent reflections
Radiation source: fine-focus sealed tube	3248 reflections with *I* > 2σ(*I*)
graphite	*R*_int_ = 0.019
φ and ω scans	θ_max_ = 25.0°, θ_min_ = 2.0°
Absorption correction: multi-scan (*SADABS*; Sheldrick, 1996)	*h* = −8→12
*T*_min_ = 0.944, *T*_max_ = 0.965	*k* = −13→13
5667 measured reflections	*l* = −12→10

### Refinement

**Table:**

Refinement on *F*^2^	Secondary atom site location: difference Fourier map
Least-squares matrix: full	Hydrogen site location: inferred from neighbouring sites
*R*[*F*^2^ > 2σ(*F*^2^)] = 0.039	H atoms treated by a mixture of independent and constrained refinement
*wR*(*F*^2^) = 0.096	*w* = 1/[σ^2^(*F*_o_^2^) + (0.0437*P*)^2^ + 0.1698*P*] where *P* = (*F*_o_^2^ + 2*F*_c_^2^)/3
*S* = 1.06	(Δ/σ)_max_ = 0.001
3787 reflections	Δρ_max_ = 0.16 e Å^−^^3^
263 parameters	Δρ_min_ = −0.24 e Å^−^^3^
1 restraint	Absolute structure: Flack (1983), 1685 Friedel pairs
0 constraints	Flack parameter: 0.14 (10)
Primary atom site location: structure-invariant direct methods	

### Fractional atomic coordinates and isotropic or equivalent isotropic displacement parameters (Å^2^)

**Table:**

	*x*	*y*	*z*	*U*_iso_*/*U*_eq_	Occ. (<1)
O1	0.95710 (16)	0.72151 (14)	0.74639 (14)	0.0404 (4)	
O2	0.97738 (18)	0.87366 (15)	0.56708 (17)	0.0462 (4)	
O3	0.8831 (2)	0.52789 (16)	0.54628 (18)	0.0454 (5)	
H3	0.930 (3)	0.488 (3)	0.520 (3)	0.056 (10)*	
P1	0.99001 (6)	0.74570 (6)	0.60864 (6)	0.03512 (16)	
C1	1.1586 (3)	0.6871 (2)	0.6245 (2)	0.0410 (6)	
C2	1.2450 (3)	0.7469 (3)	0.5653 (3)	0.0561 (7)	
H2	1.2159	0.8181	0.5184	0.067*	
C3	1.3736 (4)	0.7031 (3)	0.5743 (4)	0.0805 (11)	
H3A	1.4317	0.7456	0.5359	0.097*	
C4	1.4161 (3)	0.5965 (4)	0.6402 (4)	0.0815 (11)	
H4	1.5020	0.5657	0.6438	0.098*	
C5	1.3327 (3)	0.5355 (3)	0.7005 (4)	0.0720 (9)	
H5	1.3626	0.4641	0.7465	0.086*	
C6	1.2035 (3)	0.5802 (3)	0.6929 (3)	0.0551 (7)	
H6	1.1466	0.5386	0.7336	0.066*	
C7	0.8846 (3)	0.8021 (3)	0.9295 (3)	0.0561 (7)	
H7	0.8856	0.7192	0.9628	0.067*	
C8	0.9937 (3)	0.8099 (3)	0.8559 (2)	0.0452 (6)	
H8	0.9926	0.8912	0.8181	0.054*	
C9	1.1352 (3)	0.7835 (3)	0.9449 (3)	0.0571 (8)	
H9A	1.2009	0.7927	0.8937	0.068*	
H9B	1.1391	0.7004	0.9752	0.068*	
C10	1.1745 (3)	0.8673 (3)	1.0659 (3)	0.0697 (9)	
H10	1.1755	0.9504	1.0336	0.084*	
C11	1.0682 (4)	0.8595 (4)	1.1413 (3)	0.0788 (10)	
H11A	1.0902	0.9170	1.2145	0.095*	
H11B	1.0699	0.7791	1.1791	0.095*	
C12	0.9261 (4)	0.8856 (4)	1.0522 (3)	0.0797 (11)	
H12A	0.9222	0.9689	1.0222	0.096*	
H12B	0.8608	0.8763	1.1037	0.096*	
C13	0.7400 (3)	0.8263 (4)	0.8383 (3)	0.0776 (11)	
H13	0.7166	0.7564	0.7786	0.093*	
C14	0.627 (3)	0.8350 (16)	0.911 (3)	0.095 (5)	0.50
H14A	0.6335	0.9114	0.9559	0.143*	0.50
H14B	0.6381	0.7707	0.9749	0.143*	0.50
H14C	0.5388	0.8282	0.8467	0.143*	0.50
C15	0.714 (4)	0.938 (4)	0.748 (3)	0.108 (6)	0.50
H15A	0.7745	0.9366	0.6920	0.162*	0.50
H15B	0.7302	1.0094	0.8022	0.162*	0.50
H15C	0.6209	0.9371	0.6934	0.162*	0.50
C14'	0.635 (3)	0.7783 (17)	0.912 (3)	0.095 (5)	0.50
H14D	0.6474	0.8216	0.9944	0.143*	0.50
H14E	0.6507	0.6936	0.9306	0.143*	0.50
H14F	0.5438	0.7903	0.8562	0.143*	0.50
C15'	0.735 (4)	0.959 (4)	0.789 (3)	0.108 (6)	0.50
H15D	0.7474	1.0123	0.8642	0.162*	0.50
H15E	0.6473	0.9740	0.7266	0.162*	0.50
H15R	0.8057	0.9717	0.7467	0.162*	0.50
C16	1.3180 (4)	0.8374 (5)	1.1542 (4)	0.1122 (16)	
H16A	1.3206	0.7549	1.1833	0.168*	
H16B	1.3403	0.8899	1.2306	0.168*	
H16C	1.3831	0.8487	1.1040	0.168*	
C17	0.8687 (2)	0.6464 (2)	0.4925 (2)	0.0352 (5)	
C18	0.8980 (3)	0.6531 (2)	0.3562 (2)	0.0448 (6)	
H18A	0.8993	0.7372	0.3301	0.054*	
H18B	0.9877	0.6192	0.3638	0.054*	
C19	0.7919 (3)	0.5853 (3)	0.2483 (3)	0.0588 (8)	
H19A	0.8104	0.5973	0.1628	0.071*	
H19B	0.7987	0.4995	0.2681	0.071*	
C20	0.6479 (3)	0.6285 (3)	0.2392 (3)	0.0709 (9)	
H20A	0.6380	0.7123	0.2105	0.085*	
H20B	0.5823	0.5807	0.1733	0.085*	
C21	0.6187 (3)	0.6171 (3)	0.3728 (3)	0.0657 (9)	
H21A	0.6223	0.5326	0.3983	0.079*	
H21B	0.5272	0.6467	0.3654	0.079*	
C22	0.7219 (3)	0.6887 (3)	0.4808 (3)	0.0492 (7)	
H22A	0.7022	0.6777	0.5660	0.059*	
H22B	0.7136	0.7741	0.4590	0.059*	

### Atomic displacement parameters (Å^2^)

**Table:**

	*U*^11^	*U*^22^	*U*^33^	*U*^12^	*U*^13^	*U*^23^
O1	0.0456 (9)	0.0393 (11)	0.0388 (8)	−0.0032 (8)	0.0161 (7)	−0.0047 (7)
O2	0.0508 (11)	0.0335 (10)	0.0567 (10)	−0.0007 (8)	0.0190 (8)	0.0038 (8)
O3	0.0580 (12)	0.0339 (10)	0.0494 (11)	−0.0026 (9)	0.0233 (9)	−0.0016 (8)
P1	0.0363 (3)	0.0318 (3)	0.0396 (3)	−0.0007 (3)	0.0144 (2)	0.0000 (3)
C1	0.0396 (14)	0.0410 (14)	0.0434 (14)	0.0005 (11)	0.0132 (11)	−0.0020 (12)
C2	0.0513 (15)	0.0501 (15)	0.0746 (17)	0.0069 (17)	0.0305 (13)	0.0119 (19)
C3	0.058 (2)	0.078 (3)	0.119 (3)	0.0097 (17)	0.048 (2)	0.018 (2)
C4	0.0510 (19)	0.082 (3)	0.119 (3)	0.0225 (19)	0.035 (2)	0.012 (2)
C5	0.059 (2)	0.063 (2)	0.093 (2)	0.0203 (17)	0.0181 (18)	0.0197 (18)
C6	0.0487 (17)	0.0538 (18)	0.0643 (18)	0.0038 (14)	0.0184 (14)	0.0140 (15)
C7	0.0615 (18)	0.0665 (18)	0.0429 (15)	0.0104 (15)	0.0191 (14)	−0.0072 (13)
C8	0.0591 (17)	0.0389 (14)	0.0371 (14)	−0.0023 (14)	0.0128 (13)	−0.0048 (12)
C9	0.0543 (17)	0.065 (2)	0.0514 (16)	−0.0048 (13)	0.0134 (13)	−0.0063 (13)
C10	0.082 (2)	0.069 (2)	0.0504 (17)	−0.0200 (18)	0.0051 (16)	−0.0067 (16)
C11	0.094 (3)	0.092 (3)	0.0444 (17)	−0.004 (2)	0.0104 (18)	−0.0162 (18)
C12	0.095 (3)	0.096 (3)	0.0519 (18)	0.014 (2)	0.0266 (18)	−0.0245 (19)
C13	0.062 (2)	0.114 (3)	0.0585 (19)	0.029 (2)	0.0188 (17)	−0.015 (2)
C14	0.067 (7)	0.137 (16)	0.090 (7)	0.030 (11)	0.034 (5)	−0.024 (13)
C15	0.099 (12)	0.131 (17)	0.072 (14)	0.055 (11)	−0.012 (11)	−0.015 (12)
C14'	0.067 (6)	0.137 (16)	0.089 (7)	0.030 (12)	0.034 (5)	−0.024 (13)
C15'	0.099 (12)	0.130 (17)	0.072 (14)	0.055 (11)	−0.012 (11)	−0.014 (12)
C16	0.089 (3)	0.148 (4)	0.078 (3)	−0.027 (3)	−0.011 (2)	−0.026 (3)
C17	0.0406 (14)	0.0327 (13)	0.0352 (12)	−0.0009 (10)	0.0155 (11)	−0.0018 (10)
C18	0.0491 (15)	0.0478 (16)	0.0400 (13)	0.0045 (12)	0.0163 (12)	0.0001 (12)
C19	0.069 (2)	0.0644 (19)	0.0406 (15)	0.0064 (16)	0.0124 (14)	−0.0084 (14)
C20	0.061 (2)	0.085 (2)	0.0560 (18)	0.0042 (17)	−0.0025 (15)	−0.0180 (16)
C21	0.0420 (16)	0.081 (2)	0.070 (2)	−0.0046 (15)	0.0094 (15)	−0.0206 (17)
C22	0.0413 (15)	0.0544 (17)	0.0514 (15)	0.0028 (13)	0.0121 (12)	−0.0086 (13)

### Geometric parameters (Å, °)

**Table:**

O1—C8	1.469 (3)	C13—C15'	1.55 (5)
O1—P1	1.5853 (16)	C13—C14	1.55 (3)
O2—P1	1.4752 (18)	C13—C14'	1.57 (3)
O3—C17	1.417 (3)	C13—H13	0.9800
O3—H3	0.76 (3)	C14—H14A	0.9600
P1—C1	1.799 (3)	C14—H14B	0.9600
P1—C17	1.834 (2)	C14—H14C	0.9600
C1—C2	1.376 (4)	C15—H15A	0.9600
C1—C6	1.390 (4)	C15—H15B	0.9600
C2—C3	1.375 (4)	C15—H15C	0.9600
C2—H2	0.9300	C14'—H14D	0.9600
C3—C4	1.371 (5)	C14'—H14E	0.9600
C3—H3A	0.9300	C14'—H14F	0.9600
C4—C5	1.367 (5)	C15'—H15D	0.9600
C4—H4	0.9300	C15'—H15E	0.9600
C5—C6	1.387 (4)	C15'—H15R	0.9600
C5—H5	0.9300	C16—H16A	0.9600
C6—H6	0.9300	C16—H16B	0.9600
C7—C8	1.518 (4)	C16—H16C	0.9600
C7—C13	1.537 (4)	C17—C18	1.533 (3)
C7—C12	1.537 (4)	C17—C22	1.538 (3)
C7—H7	0.9800	C18—C19	1.522 (4)
C8—C9	1.508 (4)	C18—H18A	0.9700
C8—H8	0.9800	C18—H18B	0.9700
C9—C10	1.526 (4)	C19—C20	1.520 (4)
C9—H9A	0.9700	C19—H19A	0.9700
C9—H9B	0.9700	C19—H19B	0.9700
C10—C11	1.507 (5)	C20—C21	1.507 (4)
C10—C16	1.529 (5)	C20—H20A	0.9700
C10—H10	0.9800	C20—H20B	0.9700
C11—C12	1.513 (5)	C21—C22	1.529 (4)
C11—H11A	0.9700	C21—H21A	0.9700
C11—H11B	0.9700	C21—H21B	0.9700
C12—H12A	0.9700	C22—H22A	0.9700
C12—H12B	0.9700	C22—H22B	0.9700
C13—C15	1.53 (5)		
			
C8—O1—P1	121.25 (15)	C7—C13—C14'	107.6 (10)
C17—O3—H3	114 (2)	C15'—C13—C14'	121.0 (17)
O2—P1—O1	113.87 (9)	C15—C13—H13	106.1
O2—P1—C1	112.05 (11)	C7—C13—H13	106.1
O1—P1—C1	107.06 (10)	C14—C13—H13	106.1
O2—P1—C17	113.18 (11)	C13—C14—H14A	109.5
O1—P1—C17	102.87 (9)	C13—C14—H14B	109.5
C1—P1—C17	107.13 (11)	C13—C14—H14C	109.5
C2—C1—C6	118.6 (2)	C13—C15—H15A	109.5
C2—C1—P1	119.9 (2)	C13—C15—H15B	109.5
C6—C1—P1	121.5 (2)	C13—C15—H15C	109.5
C3—C2—C1	121.0 (3)	C13—C14'—H14D	109.5
C3—C2—H2	119.5	C13—C14'—H14E	109.5
C1—C2—H2	119.5	H14D—C14'—H14E	109.5
C4—C3—C2	119.9 (3)	C13—C14'—H14F	109.5
C4—C3—H3A	120.0	H14D—C14'—H14F	109.5
C2—C3—H3A	120.0	H14E—C14'—H14F	109.5
C5—C4—C3	120.3 (3)	C13—C15'—H15D	109.5
C5—C4—H4	119.8	C13—C15'—H15E	109.5
C3—C4—H4	119.8	H15D—C15'—H15E	109.5
C4—C5—C6	119.8 (3)	C13—C15'—H15R	109.5
C4—C5—H5	120.1	H15D—C15'—H15R	109.5
C6—C5—H5	120.1	H15E—C15'—H15R	109.5
C5—C6—C1	120.2 (3)	C10—C16—H16A	109.5
C5—C6—H6	119.9	C10—C16—H16B	109.5
C1—C6—H6	119.9	H16A—C16—H16B	109.5
C8—C7—C13	112.8 (2)	C10—C16—H16C	109.5
C8—C7—C12	108.4 (3)	H16A—C16—H16C	109.5
C13—C7—C12	113.8 (3)	H16B—C16—H16C	109.5
C8—C7—H7	107.2	O3—C17—C18	112.73 (19)
C13—C7—H7	107.2	O3—C17—C22	107.7 (2)
C12—C7—H7	107.2	C18—C17—C22	110.4 (2)
O1—C8—C9	109.9 (2)	O3—C17—P1	108.49 (16)
O1—C8—C7	107.0 (2)	C18—C17—P1	108.25 (17)
C9—C8—C7	112.7 (2)	C22—C17—P1	109.30 (16)
O1—C8—H8	109.1	C19—C18—C17	112.3 (2)
C9—C8—H8	109.1	C19—C18—H18A	109.1
C7—C8—H8	109.1	C17—C18—H18A	109.1
C8—C9—C10	112.1 (2)	C19—C18—H18B	109.1
C8—C9—H9A	109.2	C17—C18—H18B	109.1
C10—C9—H9A	109.2	H18A—C18—H18B	107.9
C8—C9—H9B	109.2	C20—C19—C18	111.4 (2)
C10—C9—H9B	109.2	C20—C19—H19A	109.3
H9A—C9—H9B	107.9	C18—C19—H19A	109.3
C11—C10—C9	109.6 (3)	C20—C19—H19B	109.3
C11—C10—C16	112.2 (3)	C18—C19—H19B	109.3
C9—C10—C16	110.6 (3)	H19A—C19—H19B	108.0
C11—C10—H10	108.1	C21—C20—C19	110.6 (3)
C9—C10—H10	108.1	C21—C20—H20A	109.5
C16—C10—H10	108.1	C19—C20—H20A	109.5
C10—C11—C12	111.8 (3)	C21—C20—H20B	109.5
C10—C11—H11A	109.3	C19—C20—H20B	109.5
C12—C11—H11A	109.3	H20A—C20—H20B	108.1
C10—C11—H11B	109.3	C20—C21—C22	111.4 (3)
C12—C11—H11B	109.3	C20—C21—H21A	109.3
H11A—C11—H11B	107.9	C22—C21—H21A	109.3
C11—C12—C7	113.0 (3)	C20—C21—H21B	109.3
C11—C12—H12A	109.0	C22—C21—H21B	109.3
C7—C12—H12A	109.0	H21A—C21—H21B	108.0
C11—C12—H12B	109.0	C21—C22—C17	110.7 (2)
C7—C12—H12B	109.0	C21—C22—H22A	109.5
H12A—C12—H12B	107.8	C17—C22—H22A	109.5
C15—C13—C7	119.6 (14)	C21—C22—H22B	109.5
C7—C13—C15'	108.0 (13)	C17—C22—H22B	109.5
C15—C13—C14	103.0 (17)	H22A—C22—H22B	108.1
C7—C13—C14	115.1 (11)		

### Hydrogen-bond geometry (Å, °)

**Table:**

*D*—H···*A*	*D*—H	H···*A*	*D*···*A*	*D*—H···*A*
O3—H3···O2^i^	0.76 (3)	1.94 (3)	2.695 (3)	170 (3)

Symmetry codes: (i) −*x*+2, *y*−1/2, −*z*+1.
